# Micro-CT evaluation of canal shaping extent with apex locator–integrated endodontic motors: An in vitro study

**DOI:** 10.1371/journal.pone.0355939

**Published:** 2026-08-27

**Authors:** Zainab Shirazi, Anas Al-Jadaa, Dalya Sharaf, Abdulrahman Mohammed Saleh, Ahmed Jamleh

**Affiliations:** 1 Department of Clinical Sciences, College of Dentistry, Ajman University, Ajman, United Arab Emirates; 2 Centre of Medical and Bio-allied Health Sciences Research, Ajman University, Ajman, United Arab Emirates; 3 Department of Restorative Dentistry, College of Dental Medicine, University of Sharjah, Sharjah, United Arab Emirates; Danube Private University, AUSTRIA

## Abstract

**Introduction:**

This in vitro study evaluated the extent accuracy of canal shaping to apical constriction (AC) with electronic apex locator (EAL)-integrated endodontic motors using micro-computed tomography (MicroCT).

**Materials and Methods:**

Sixty single-canalled teeth were decoronated, scanned using microCT, and distributed into three groups: Rooter Universal, Tri Auto ZX II, and control group (n = 20 each). The working length (WL) was determined simultaneously during canal shaping using the auto-reverse function set at the 0.5 mark in the RU and TA groups. In the control group, the WL was measured before the shaping using Root ZX (J. Morita, Tokyo, Japan). In the three groups, canals were shaped using ProTaper Gold files (Dentsply Sirona, Ballaigues, Switzerland) until the preset mark was reached in the two experimental groups and to the measured WL in the control group. Afterwards, post-shaping microCT scans were taken. Scan superimposition was conducted to assess the absolute distance between the final shaping endpoint and AC. Negative and positive data were recorded when the measurements were found short or beyond the AC, respectively. Analysis of variance (ANOVA) and post-hoc Tukey tests were used to analyze the distance. The chi-square test was applied to analyze the accuracy of the measurements with respect to the AC. The significance level was set at 5%.

**Results:**

No over-shaping beyond the apical foramen was found in any sample, and all systems showed similar accuracy when a tolerance of 0.5 mm was set ((V = 0.263, P = 0.126). However, the shaping extent to AC was closer in the control group than the RU (P = 0.001) and TA (P < 0.001), while RU and TA were similar (P = 0.588).

**Conclusions:**

The accuracy of WL determination was not affected by the tested EAL. However, the standalone EAL was more reliable at maintaining AC integrity.

## Introduction

The success of root canal therapy hinges critically on establishing the proper working length (WL). Research indicates that when preparation and filling remain within the boundaries of the root canal system, patients experience significantly better long-term outcomes [1]. Practitioners face significant challenges navigating the complex and variable root canal anatomy while aiming to achieve thorough three-dimensional shaping without compromising critical apical morphology, especially the apical constriction (AC), particularly when relying on conventional radiographic or tactile methods alone. However, the use of electronic working length determination (EWLD) alone addresses many of these limitations by allowing for accurate measurement as well as precise preparation of the apical portion [2]. Achieving the appropriate balance between adequate shaping and preservation of apical anatomy remains a fundamental objective in endodontics, as over-obturation carry well documented clinical consequences. The American Association of Endodontists describes the AC as the canal’s narrowest diameter near its exit point [3]. This landmark exists consistently across all root canals and can be identified using the EAL in daily practice [4,5].

The EAL works by connecting it to the patient’s lip and inserting a file into the canal. It can identify the point where the root canal space connects to the periodontium, thereby establishing the endpoint for shaping and obturation.

It has been demonstrated that the accuracy of EALs in identifying the correct WL reaches 100% [6]. However, the WL might differ throughout the treatment and should be re-measured before root canal filling to avoid procedural errors [6]. This clinical reality prompted the development of EAL-integrated endodontic motors, which shape canals while receiving continuous feedback from the EAL. This integration eliminates the need for repeated WL calibration and addresses potential alterations in WL during later treatment stages, making them reliable alternatives to conventional techniques, particularly in curved canals [8,9].

The accuracy of endodontic motors with integrated EALs for continuous length control has been studied ex vivo and in vivo at specific stages by measuring the distance from the file tip to an anatomical landmark with or without grinding the apical root using different tools such as dental radiography [10], scanning electron microscopy [11], and stereomicroscopy [8].

While these methodologies have been efficaciously used for years, they were considered invasive and inadequate for analyzing the spatial relationship between the shaping boundary and the anatomic structures of the apical region [12]. Furthermore, considering the varying apical anatomy, micro-computed tomographic (MicroCT) assessment was introduced as a non-invasive tool for precise measurements which can accurately detect the location of the AC and evaluate the precision of the EALs [4,12–15].

Evaluating the performance capabilities of commercially available EAL-integrated endodontic motors during the canal shaping may provide valuable insights regarding their ability to maintain the AC throughout the entire shaping process. Therefore, the current study assessed two endodontic motors (Rooter Universal “RU” and Tri Auto ZX II “TA”) integrated with EALs for continuous length control in comparison to conventional length determination to AC. The null hypothesis tested here was that the tested EAL–integrated endodontic motors allowed canal shaping while maintaining AC integrity.

## Materials and methods

Ethical approval was obtained from the Research Ethics Committee at Ajman University (D-H-F-6-Jan, Date: 8.2.2022). The study included single-rooted teeth chosen from a pool of recently extracted teeth in the university dental clinics.

### Sample size calculation

The sample size was determined based on a previous study [16] using G*Power 3.1.9.7 (Heinrich-Heine-Universität Düsseldorf, Germany). It was calculated using a 0.05 significance level and 0.80 power to detect a minimum distance difference of 0.9 mm (Distance from the file tip to the AC) between the tested groups. The result reported that a minimum of 18 teeth per group was required. To enhance the robustness of the study, the sample size was increased to 20 teeth per group.

**Fig 1:**
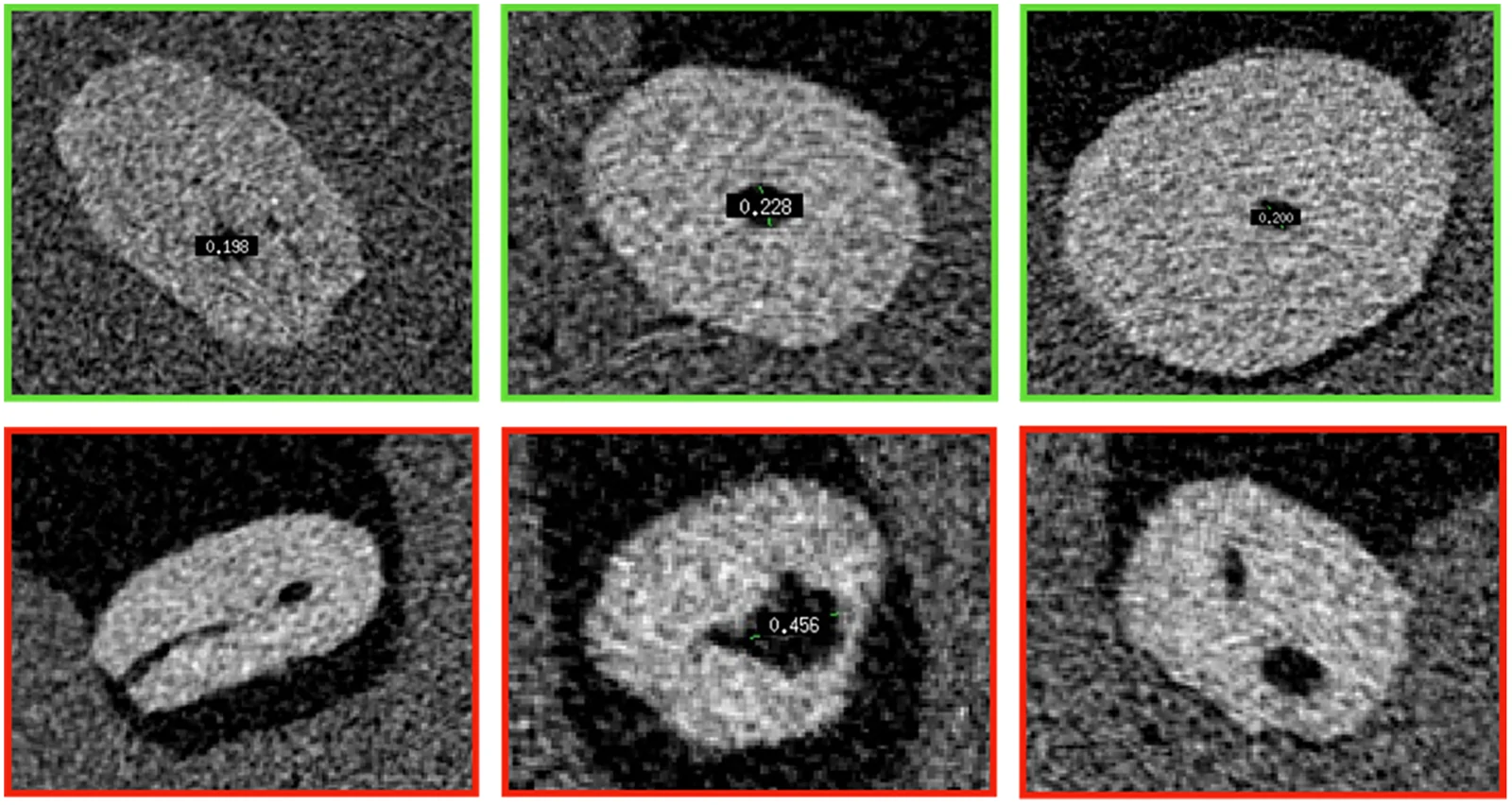
Micro-CT scans in the axial view showing examples of included teeth (top row) and excluded teeth (bottom row).

The study started on the 1^st^ of March 2022 with experimental samples selected from a teeth pool collected within 6 months prior to the study start date. The teeth collection was anonymous with no identifiable personal information and for reasons not related to the study. General informed consent was obtained from all patients to allow the use of their extracted teeth in research projects. Periapical radiographs were taken to select teeth with mature roots, single canals, and patent apical foramina. After meticulous cleaning with an ultrasonic scaler, teeth were rinsed with a 3% hydrogen peroxide solution and then stored in a 0.9% saline solution at 5°C in a refrigerator. The initially selected samples were then placed in putty impression material (Hydrorise; Zhermack, Badia Polesine, Italy) and were further imaged using a micro-CT scanner at low resolution (Scanco µCT 100; SCANCO Medical, Brüttisellen, Switzerland), to evaluate and standardize anatomical parameters and select appropriate teeth matching the pre-determined criteria (Fig 1)

### Sample Preparation

**Fig 2:**
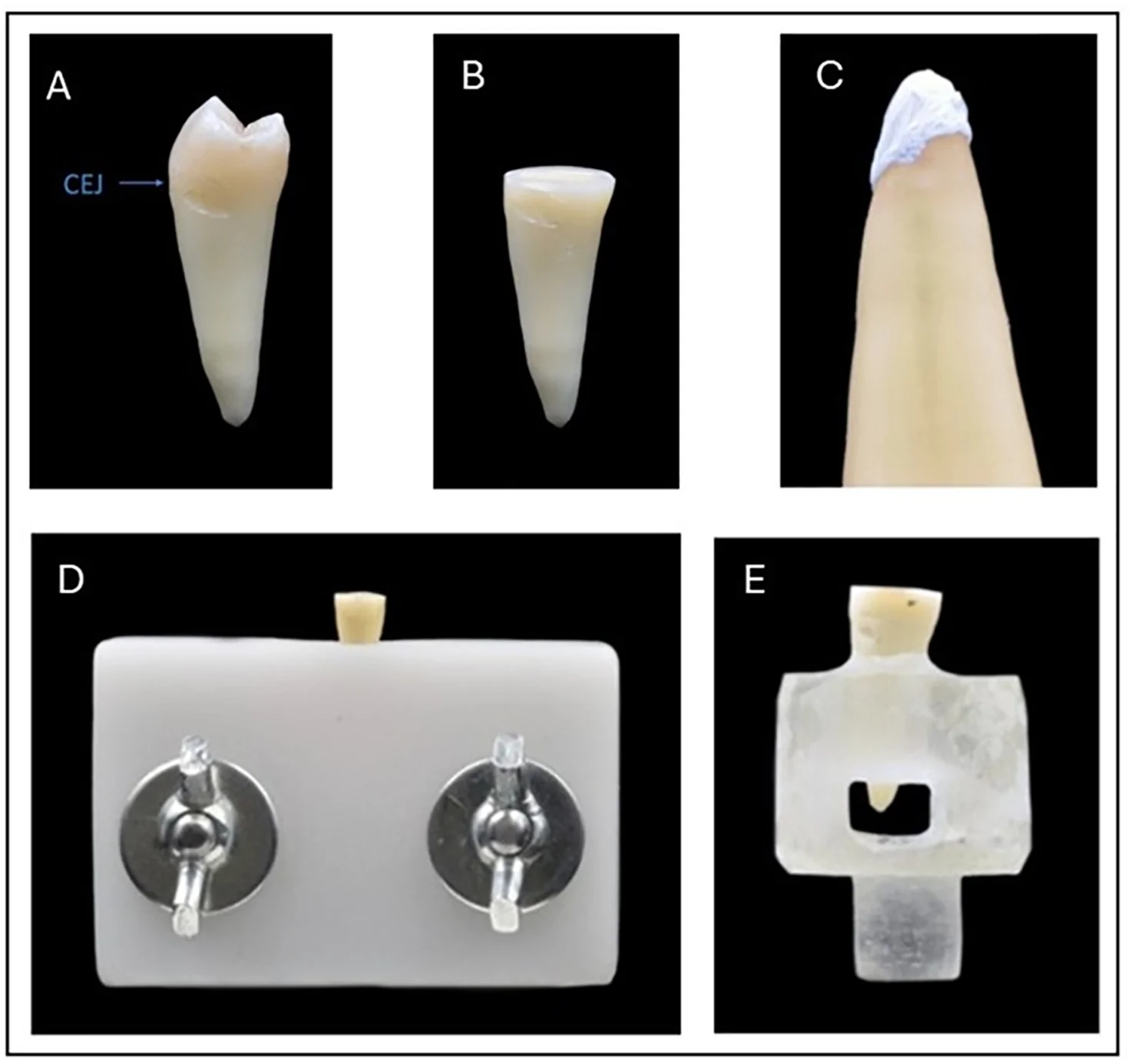
Tooth sectioning and embedding. (**A)** Un-sectioned tooth with the CEJ (cementoenamel junction) marked. (**B)** Sectioned Tooth. (**C**) Apex Covered with Teflon tape**. (D**) Custom-made mold for sample fabrication with tooth positioned inside. (**E**) Final embedded sample.

The teeth were decoronated at 2 mm coronal to the cementoenamel junction (Fig 2, A) using a diamond disc to create a sample with horizontal flat surface that would facilitate easier access with a reliable reference point (Fig 2, B).

The teeth were then mounted in self-curing acrylic resin after securing the apical foramen with a Teflon band (Fig 2, C). The mounting took place within a custom-made mold featuring an apical window for communication with the apex via normal saline fluid (Fig 2, D & E). After the access cavity was established, apical patency was confirmed under magnification with a size-10 finger spreader (Dentsply Sirona, Ballaigues, Switzerland) passively introduced to the canal until it was visible at the apex.

**Fig 3:**
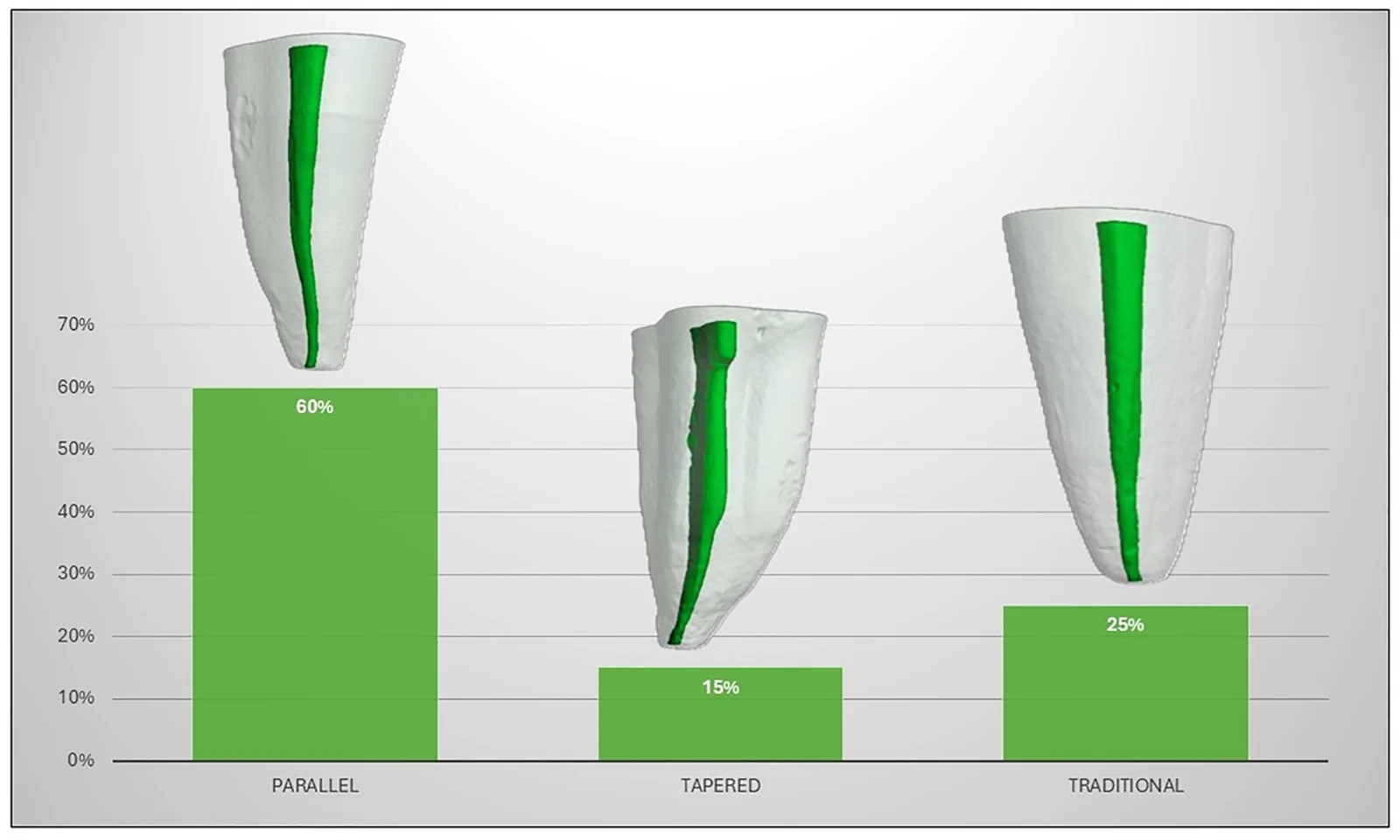
Micro-CT images of topography types and their percentage presentation.

**Fig 4:**
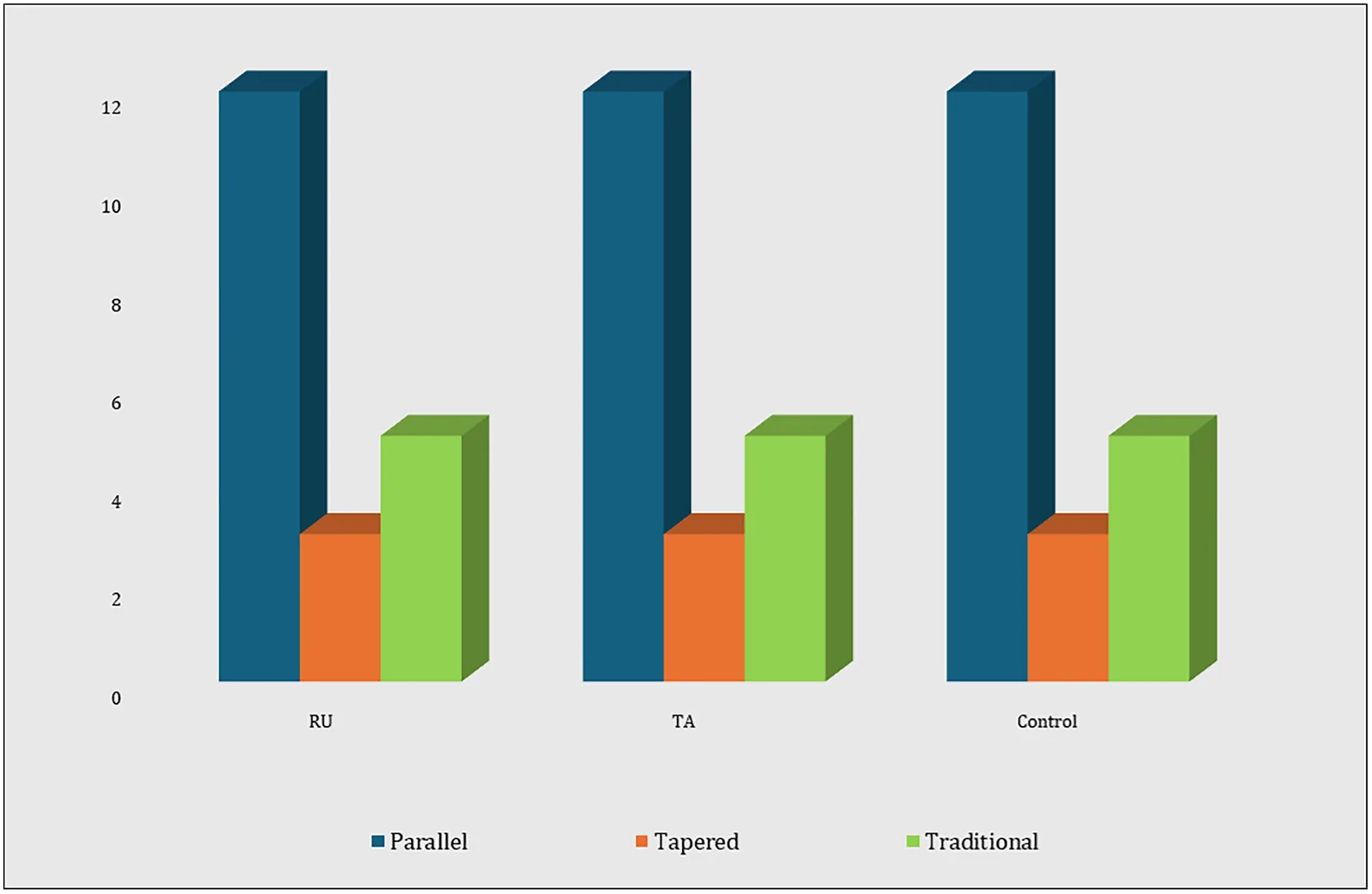
Bar chart showing distribution of topography types across study groups.

**Fig 5:**
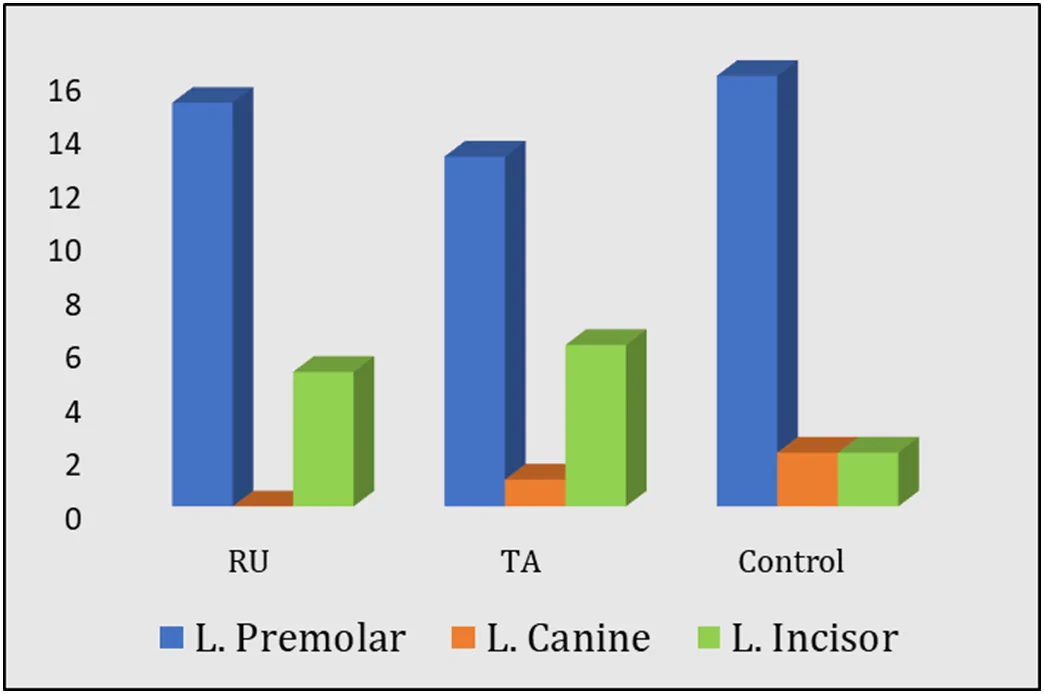
Bar chart showing distribution of tooth types across study groups.

The region of interest was limited to the apical 5 mm of each sample, which was scanned again with the micro-CT scanner at 90 kV and 200 µA with an isotropic resolution of 20 µm. Using an µCT evaluation program (SCANCO Medical AG) on axial view, each sample was inspected to determine the diameter of the narrowest zone representing the AC. From the sagittal view, the apical constriction topography type was determined (Fig 3). In addition, the distance from the lower margin of the constriction to the root apex and apical constriction diameter were considered. Based on these measures, the teeth were randomly and equally distributed into RU, TA, and control groups (n = 20 each) (Table 1) (Fig 4). Tooth type distribution across the study groups (Fig 5) was excluded as a sampling parameter, given its comparatively limited impact on apex locator accuracy compared to apical constriction diameter and topography.

**Table 1:** The diameter of the apical constriction and its distance to the major apical foramen before canal shaping.

Group	Diameter (Mean±SD) (mm)	Distance (Mean±SD) (mm)
**Rooter Universal**	0.23 ± 0.05	0.80 ± 0.34
**Tri Auto ZX II**	0.24 ± 0.06	0.68 ± 0.26
**Control**	0.26 ± 0.06	0.76 ± 0.29
**P value**	0.20	0.42

### Experimental Procedures

**Fig 6:**
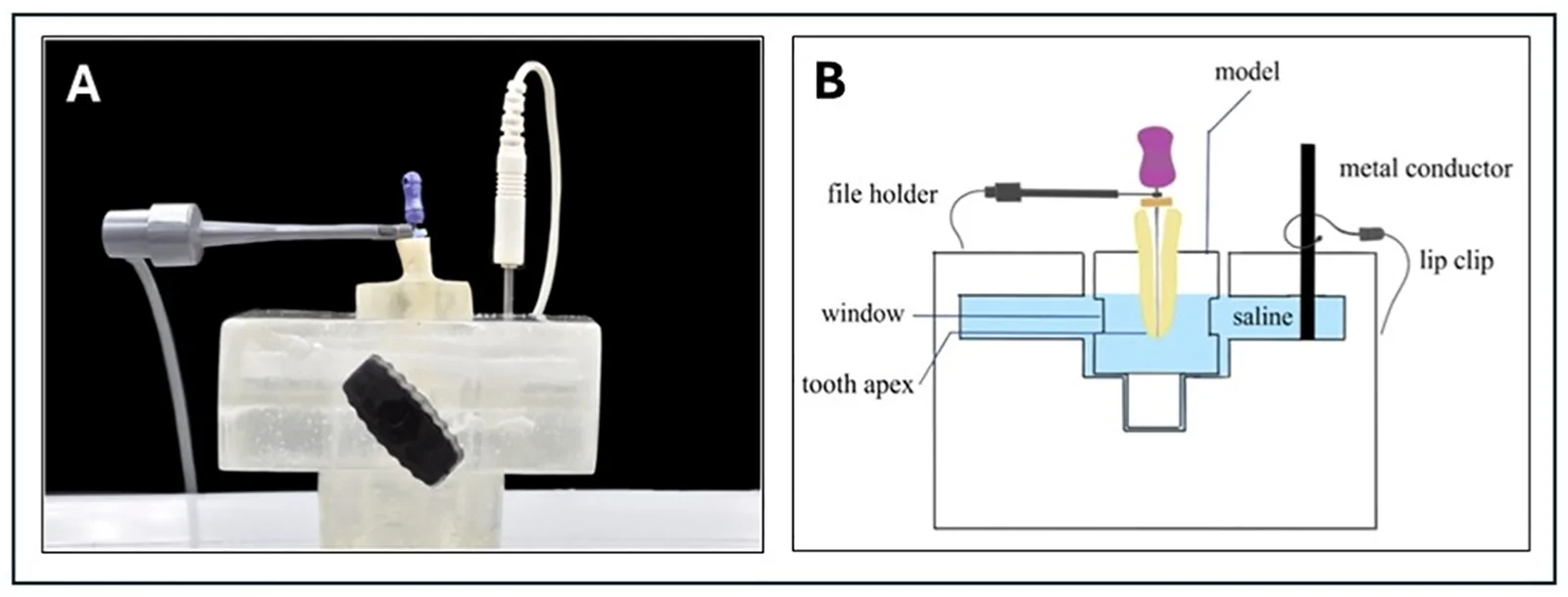
Root Apex simulation model. **(A)** Custom-made device which allowed electrical conductivity and completion of circuit. (**B)** Diagrammatic representation of apex locator simulation set-up.

A custom-made device (Fig 6) was fabricated with a slot for saline into which the tooth-mounted model was placed. The device included a metal conductor onto which the metal lead of the apex locator device and motors could be fixed. 0.9% saline solution was used as a conducting medium to simulate *in vivo* conditions. The windows in the tooth-mounted sample allowed for free flow of saline and completion of the circuit when a file was introduced to the apical foramen.

In RU and TA groups, all apical control functions were set to the 0.5 mark, and WL control during shaping was maintained through the auto-reverse function.

In the control group, Root ZX (J. Morita, Tokyo, Japan) was connected to a size 10 spreader, which was gently inserted into the root canal, flooded with 1.3% NaOCl, until the numeric display of the EAL flashed “apex.” The file was then pulled out gradually until the reading reached the final green bar. The measurements were considered valid if the reading/signal on screen remained stable for at least 5 seconds, and triple-checked to ensure accuracy. Three rubber stoppers were adjusted to the reference surface, and the spreader was measured carefully to the nearest 0.01 mm with the aid of a digital caliper (Mitutoyo, Aurora, IL; USA). This length was used for canal shaping with an endodontic motor (X-Smart Plus; Dentsply-Maillefer, Ballaigues, Switzerland).

Canal shaping was carried out by a trained single operator who was blinded to the MicroCT assessment and calibrated to use the devices following the manufacturers’ instructions. All canals were shaped using ProTaper Gold (Dentsply Sirona Endodontics, Ballaigues, Switzerland) until F3, F4, or F5 based on the AC diameter (Smaller than 0.20 mm, between 0.20 and 0.30 mm, or larger than 0.3 mm, respectively). During shaping, 1 ml of 1.3% NaOCl solution was delivered after each file with a 30-gauge side-vented needle (CERKAMED Medical; Stalowa Wola – Poland), and patency was checked by inserting a size 10 finger spreader to the WL. Then, the canal was rinsed with 5 ml of 17% EDTA, followed by a final wash with 1 ml of normal saline solution.

All EALs were calibrated before use according to the manufacturers’ instructions, and measurements were taken with the devices fully charged. The EALs were used in a randomized fashion to avoid bias.

### MicroCT Analysis

**Fig 7:**
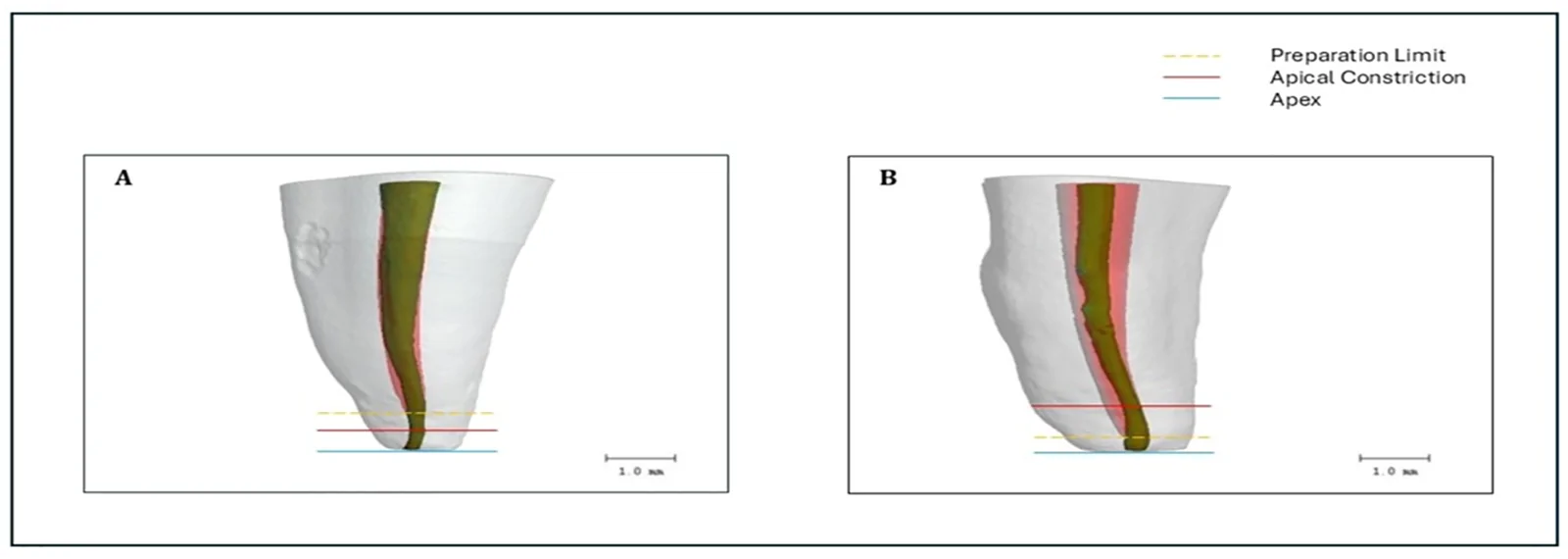
Pre- (green) and post-scans (red) of samples superimposed showing. **(A)** Preservation. (**B**) Non-preservation (apical constriction, preparation limit and apex marked).

The apical 5 mm of the samples were MicroCT scanned again with the same parameters. Post-shaping scan was superimposed on the pre-shaping counterpart using the evaluation program and then imported into ImageJ (Fiji, v.1.54c; Madison, Wisconsin, USA) to quantitatively evaluate AC preservation by measuring distance (mm) from the end point of shaping to the AC. The distance was given a negative value if it was short of the AC and a positive value if it extended apical to it. The assessor, who was blinded to the tested EALs, measured the distance 3 times, and the average was calculated (Fig 7). Measurement reproducibility for the 3 readings was assessed using intraclass correlation coefficient (ICC).

### Statistical analysis

Since the data were normally distributed (Shapiro-Wilk test, P > 0.05), and the homogeneity of variances was met (Levene’s test, F(2, 57) = 1.319, P = 0.276), analysis of variance (ANOVA) and post-hoc Tukey tests were used to analyze the distance in the tested groups. The frequencies shown from +0.5 to –0.5 mm away from the AC were considered as accurate, whereas the other frequencies were labelled as inaccurate. These categories were compared using a chi-square test in the tested groups. The significance level was set at 5%.

## Results

Table 2 shows the mean distance measurement to AC,the accuracy of detecting the AC within a tolerance of 0.5 mm, and the final file size used to shape the canalsfor each group. Shaping reached the AC in 8 samples of the control group, compared to 1 sample in RU and none in TA. Overshaping was observed in 7 samples in the control group, 1 sample in the TA, and none in RU, while undershaping was found in 5, 19, and 19 samples in the control, TA, and RU, respectively.

**Table 2:** Distance measurement to apical constriction,accuracy of detecting the apical constriction within a tolerance of 0.5 mm, and the final file size used to shape the canals in each group.

Group	Mean±SD (mm)	Accurate N (%)	Final File Size		
			F3	F4	F5
**Rooter Universal (n = 20)**	0.58 ± 0.30^a^	9 (45%)	5	13	2
**Tri Auto ZX II (n = 20)**	0.70. ± 0.42^a^	8 (40%)	7	12	1
**Root ZX (Control) (n = 20)**	0.11 ± 0.42^b^	14 (70%)	4	12	4
**Statistical analysis**	ANOVA and Tukey tests	Chi-square test	Chi-square test		
**P value**	<0.01	0.126	0.572		

Different letters indicate the presence of a statistical difference

The triplicate measurements demonstrated excellent reliability (average-measures ICC = 0.997, 95% CI: 0.997–0.999). In the control group, the shaping extents reached 0.11 [±0.42] mm which was found significantly closer to the AC, compared to the RU (0.58 [±0.30] mm; p = 0.001) and TA (0.70 [±0.42] mm, respectively (F(2, 57) = 13.150, P < 0.001, ƞ^2^ = 0.316). The shaping extent to AC was closer in the control group than the RU (P = 0.001) and TA (P < 0.001), while RU and TA were similar (P = 0.588). Moreover, when considering the tolerance limit of 0.5 mm, although the control group demonstrated a numerically higher measurement accuracy rate (70%) compared with the RU (45%) and TA (40%) groups, these differences did not reach statistical significance (V = 0.263, P = 0.126), Nonetheless, no over-shaping beyond the apical foramen was found in any sample.

## Discussion

It is documented that a 2D radiograph is inadequate for determining working length, and the EAL is preferred because it provides readings close to the AC [2]. The present in vitro study analyzed the accuracy of two EAL-integrated endodontic motors (RU and TA) for continuous length control to the AC in comparison to standalone EAL. The distance measurements revealed that the WL used for shaping in the control group was significantly closer to the AC, compared to those in RU and TA. Also, the control group showed higher accuracy than the RU and TA. Therefore, the null hypothesis was rejected.

Precise measurement of WL is essential to preserve the apical anatomy and conduct shaping within the root canal system. Past experiments have considered different apical landmarks to set WL and assess the accuracy of EALs, such as the radiographic apex [10], apical foramen [8,17,18], and AC [19–22]. In this study, the AC was chosen because the ESE [5] considers the AC the ideal endpoint of canal shaping. Previous studies showed that determination of AC is possible using EAL with high accuracy that is not influenced by tooth type, root canal type, status of the periapex, or clinical condition in primary and permanent teeth [2,4,5,7,14,23].

The AC was observed in all samples, which was also evident in a previous anatomical study [4]. The AC was found 0.747 ± 0.296 mm coronal to the apical foramen. This was located slightly more coronal than those observed in previous studies [4,24,25]. This discrepancy might be explained by the type and age of teeth included and the methodological variations.

Setting the reference point for EAL measurements at a consistent location is a requirement to reduce the risk of canal shaping errors [6,25–27]. In this experiment, following the manufacturers’ instructions for AC location, the 0.5 mark was selected for all tested EALs. However, the variability in AC and apical foramen positions across different teeth can present challenges, and the 0.5 mark has been considered an arbitrary indicator of the file position [12,25,28]. Thus, this study established the tolerance range at 0.5 mm as shown in most in vivo studies [28–31].Favorably, microCT scans showed that none of the samples had the WL exceeded the root apex. With respect to the AC, the solitary determination of WL using the standalone EAL resulted in superior accuracy compared to the continuous length control employed in EAL-integrated endodontic motors. However, previous studies showed that the latter revealed better accuracy [2,8,17,18,21,22].

The observed differences in the mean distances between the apical foramen and the AC across groups, ranging from 0.68 to 0.80 mm, may appear numerically modest and arguably within the range of inherent anatomical variation of the apical region. Similarly, the absolute millimetric differences in shaping extent between groups might not seem immediately striking. However, a critical distinction must be made between acceptable clinical performance and real procedural outcome when interpreting these findings.

For verification of the study outcome, two variables were considered: the accuracy of EALs in locating the AC, and preserving the integrity of the AC after instrumentation. Regarding the first variable, EALs demonstrated broadly acceptable performance across all test groups, as the recorded distances fell within ranges commonly considered clinically tolerable in routine endodontic practice. However, when the second variable is examined, a meaningful dissociation becomes apparent. The shaping extents in the RU and TA groups were statistically significantly farther from the AC compared to the control group, indicating that the electronically determined working length was not consistently translated into an equivalent shaping outcome. The mean shaping extents could represent a mixture of short and extended preparations, averaging out. It is worth noting that shaping reached the AC in 40% of the control samples, compared to 5% in RU and none in TA. Overshaping was observed in 35% of the control group, 5% of the TA, and 0% in RU, while undershaping was found in 25%, 95%, and 95% in the control, TA, and RU, respectively. Relative to AC, the direction of the shaping (undershaping vs. overshaping) has distinct clinical consequences. This gap between acceptable diagnostic performance and real procedural result is precisely what confers clinical relevance to the present findings. A shaping deviation of 0.5–0.7 mm from the apical constriction, although small, can under certain conditions make root canal obturation difficult after instrumentation. This may lead to problems such as over‑obturation, particularly in treatments performed by less experienced clinicians. Therefore, even statistically modest differences in shaping extent should not be dismissed as clinically inconsequential, as in endodontics, the apical few tenths of a millimetre carry a disproportionate biological and clinical effect on the treatment outcome.

The control group demonstrates the best mean performance (0.11 mm from AC) but also exhibits a standard deviation (±0.42 mm) equal to that of the TA group and larger than the RU group (±0.30 mm). While the standalone EAL produces better average accuracy, it does not necessarily produce more consistent results. The differences observed between the standalone EAL and the EAL-integrated motor groups can be attributed to several interrelated factors. Although all systems employ impedance-based electronic working length determination, they fundamentally differ in their operational mode. The standalone EAL permits a controlled, static measurement with operator-verified signal stability, whereas the integrated systems must perform continuous length monitoring concurrently with active rotary instrumentation. This can introduce an inherent electromechanical latency between the moment the EAL detects the preset threshold and the moment the motor physically reverses direction. During this latency window, the file continues to advance apically under rotational momentum, displacing the effective shaping endpoint beyond the intended position. The lag is caused by signal processing time, interruption of motor commands, and mechanical inertia — factors that are entirely absent in the static measurement protocol of the standalone EAL. Additionally, the 0.5 mark threshold setting provides a narrow safety margin against the screw in effect of the file that may insufficiently accommodate the motor’s response time [32], and signal variability during active instrumentation — arising from file rotation, variable canal wall contact, and fluctuating irrigant presence — may further impair consistent threshold detection in the integrated systems. These combined factors account for the significantly greater distance from the AC observed in the RU and TA groups compared to the control.

MicroCT was used to perform pre- and post-shaping scans and superimpose them in exact locations, eliminating the interference of metallic instruments that could create artifacts and affect reading accuracy. Additionally, the current analysis used the most apical portion of the AC as a reference point for comparison with EAL measurements. Integrated devices might experience a delay between determining the AC location and stopping further file advancement under auto-reverse function [33], possibly resulting in slight over-shaping. Meanwhile, the measured WL with the standalone EAL provided a more gradual and controlled WL measurement [34]. Another possible explanation is the low setting of the 0.5 mark which might overstrain the response time of the auto-reverse function [31]. Both issues highlight the importance of utilizing a standalone EAL to maintain accurate measurements.

The in vitro results cannot be directly extrapolated to clinical practice because there are some inconsistencies in EAL measurements, even in critically controlled conditions. For example, age of the patient, status of the pulp and apical tissue, canal morphology, metallic restorations, and bleeding could alter the function of EALs and act as sources of heterogeneity [4,7,22].

The present study lies in determining the AC which is fundamental for both biological and technical objectives in non-surgical root canal treatment. However, it represents one element within a complex of factors that collectively impact the treatment outcome. The Kiviat diagram [35] illustrates this multifactorial concept, in which apical health is not determined by a single parameter but by the interaction among multiple factors, including the extent of root canal obturation, its homogeneity, and the quality of the coronal restoration. This diagram shows that the worst outcomes are associated with the cumulative presence of multiple deficiencies. This highlights that endodontic success is influenced by multiple factors rather than just the apical area, and that focusing only on the apical constriction can oversimplify the biological and technical needs for reliable healing.

In this study, there are certain limitations that should be considered. Despite the variations in the morphology of natural teeth, attempts were made in the present study to ensure comparability of the experimental groups. Therefore, the samples in all groups were balanced with respect to the AC diameter and distance between the AC and apical foramen, obtained from the microCT baseline scans. Besides that, canal configuration, coronal flaring, types and amount of irrigants, and shaping system were standardized across all samples. Furthermore, the hand movement during canal shaping might be uncontrolled and could introduce bias or inaccuracies to the data generated. However, the operator was an expert in Endodontics who carefully conducted the experiment using a consistent and gentle shaping procedure to minimize operator error. The current study used single-rooted teeth, without periodontal ligament or pulpal tissue, which were prepared with one file system. Further research is needed to validate these findings with additional devices, file systems, and clinical conditions. An additional limitation of the current study is the asymmetry between the control protocol and the integrated motor groups determining the WL. The control condition relied on a triple‑verified, static EAL measurements, which provides an unusually precise reference point and may therefore represent an optimistically ideal baseline. This level of verification is not typically implemented in routine clinical practice, and the discrepancy should be considered when interpreting the comparative performance of the integrated systems.

## Conclusions

Under the current in vitro conditions, a standalone EAL exhibited higher accuracy in length determination compared to the EALs integrated in the tested endodontic motors. Judicious clinical use of EAL integrated motors might be advisable.
